# Smile Aesthetic Evaluation on Videographs: An Intra-Rater and Inter-Rater Agreement Study

**DOI:** 10.3390/dj10050087

**Published:** 2022-05-16

**Authors:** Mathias Faure-Brac, Angéline Antezack, Sebastien Melloul, Mehdi Hadj Saïd, Anne Raskin, Virginie Monnet-Corti

**Affiliations:** 1 Faculté des Sciences Médicales et Paramédicales, Ecole de Médecine Dentaire, Aix-Marseille Université, Provence-Alpes-Côte D’azur, 13385 Marseille, France; mathias.faure-brac@univ-amu.fr (M.F.-B.); angeline.antezack@univ-amu.fr (A.A.); dr.melloul@gmail.com (S.M.); mehdi.hadj@yahoo.fr (M.H.S.); anne.raskin@univ-amu.fr (A.R.); 2 Assistance Publique-Hopitaux de Marseille (AP-HM), Hopital Timone, Provence-Alpes-Côte D’azur, 13385 Marseille, France; 3 UMR D-258 Microbes Evolution Phylogénie et Infection (MEPHI), Institut de Recherche Pour Le Développement (IRD), Aix-Marseille Université, Provence-Alpes-Côte D’azur, 13385 Marseille, France; 4 UMR 7268 Anthropologie Bio-Culturelle Droit Éthique et Santé, Aix-Marseille Université, Provence-Alpes-Côte D’azur, 13385 Marseille, France

**Keywords:** aesthetics, smiling, score, periodontics, videography

## Abstract

The aim of this study was to assess on videographs the intra- and inter-rater reproducibility of the Smile Esthetic Index (SEI) that has been previously validated on photographs. Smile videographs were obtained using a smartphone associated with the Smile Lite MDP mounted on a tripod. They were then randomized and evaluated twice consecutively at a 1-week interval by three periodontists according to the SEI based on 10 variables. Cohen’s Kappa and Fleiss’ Kappa tests were performed to measure intra- and inter-rater agreement. Sixty-five smile videographs of 24 men and 41 women (mean age 33 ± 11.3 years) were scored. A mean intra-rater agreement of 0.68 (0.64–0.73) was obtained, representing substantial agreement. The inter-rater agreement calculated for each variable ranged from 0.31 for the variable “absence of visible excessive gingiva” to 0.90 for the variable “absence of diastema and/or missing inter-dental papilla.” Within the limits of this study, we have demonstrated that it was possible to use videographs to reproducibly evaluate an aesthetic score (SEI) previously validated on photographs.

## 1. Introduction

The smile of patients is a sign of their satisfaction at the end of the treatments but also of their complexes before care. Several studies have shown that the aesthetics of the smile are a major concern for patients and have a significant impact on facial attractiveness [1,2,3]. Recently, the Smile Esthetic Index (SEI) has been proposed as a reliable and reproducible method to evaluate the aesthetics of a smile using photographs [4,5]. However, the visibility of the periodontium inevitably varies depending on whether the smile is “natural” or “forced” and thus appears difficult to assess objectively on photographs [6,7]. Indeed, when the dentist asks the patient to perform a forced smile in front of the camera lens, the patient displays less periodontium than the actual maximum smile [8].

In plastic and reconstructive surgery, studies [9,10] have evaluated the smile dynamics and soft tissue changes that occur as the face transitions from the resting to the maximum smile position. These studies have shown that the evaluation of the smile should be done on a dynamic capture of this mimic rather than on static captures. Using videographs, Tarantili et al. [11] showed that the average duration of a spontaneous smile was 500 ms, which explains the intrinsic difficulty of capturing this very brief moment on photographs. However, the patient and his family will judge the aesthetic results of periodontal treatments when the maximum of gum is exposed even though the maximum smile lasts a very short time [12]. Walder et al. [13] compared the use of videographs and photographs to assess the aesthetics of the natural and forced smile. They concluded that videographs provided diagnostic information that cannot be obtained with photographs alone, and also that videographic images should be preferred to still images by professionals. Currently, videographs are used in restorative or prosthetic dentistry as well as in orthodontics to evaluate teeth and their aesthetics during the smile [14,15,16,17]. In contrast, in periodontics, gingival aesthetics have been assessed primarily on photographs [5,12]. A recent review of the literature concerning the evaluation of gingival aesthetics after root coverage concluded that recording short videographs before and after surgery rather than using photographs could more accurately unveil the visibility of the periodontium during smiling and speaking and therefore would allow a better aesthetic evaluation of the results [18].

The aim of this study was to assess on videographs the intra- and inter-rater reproducibility of the Smile Esthetic Index (SEI) that has been previously validated on photographs.

## 2. Materials and Methods

This study was approved by our university hospital (Assistance Publique—Hôpitaux de Marseille, AP-HM), under the registration n° 2019-106. An informed consent statement authorizing the anonymous use of the videographs was obtained for each patient.

Patients were selected over a time period of 6 months (from 1 January 2019 to 30 June 2019) according to the following inclusion criteria:

− Age > 18 years.
− Not belonging to a “protected patient” category.
− Healthy and/or reduced periodontium.
− Full dental arch in the maxilla (at least 15 to 25).
− Coming for a consultation at the periodontology department, AP-HM.

The calculation of the sample size (*n* = 65) was undertaken only to validate on videographs the feasibility of using a score already validated on photographs [4].

Only one investigator (the resident) recorded, edited and numbered the videographs. Three raters (one resident, two teachers in Periodontology) evaluated the videographs according to the SEI (Rotundo et al., 2015).

### 2.1. Equipment for the Acquisition of Videographs and for Their Editing, Storage and Viewing

A smartphone (Iphone 8 ©, Apple, Cupertino, CA, USA) was used for the acquisition of videographs. Its lens was positioned in the middle of the Smile Lite MDP ©, (Smile Line, St-Imier, Switzerland), which allows a reproducible condition of light, and both were mounted on a 160 cm tripod with a 360° 3D swivel head (AMZDEAL tripod Camera 160 cm ©, AMAZON, Seattle, WA, USA).

The editing software (Imovie ©, Apple) allowed us to keep the most relevant moments of the videographic sequences.

The videographs were in MPEG-4 format with a resolution display of 1920 × 1080 pixels.

An IPad 2 © (Apple) and a Macbook Pro © (Apple) computer were used to collect the data and to view the videographs.

### 2.2. Conducting the Standardized Videography

#### 2.2.1. Position of the Investigator

Sitting in an operator’s chair behind the lens at the same height and facing the subject (front position).

#### 2.2.2. Position of the Subject Being Evaluated

At 35 cm from the camera lens, sitting on an operator’s chair with the back against the backrest in a straight position, the two feet on the ground, and the bi-pupillary plane parallel to the ground.

After the frontal shot, the investigator rotated the seat so that the subject was in profile (side position) and then ¾ positions [10,15].

### 2.3. Realization of the Shooting: Scenography

**Figure 1:**
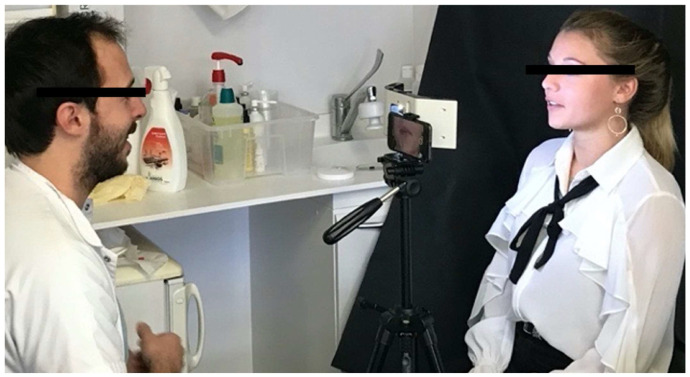
Set-up of the acquisition system for recording a videograph.

To obtain and capture a natural smile but also a large spontaneous smile and laughter, the scenography consisted of 3 steps: (Figure 1)

1. Confidence building and relaxation of the subject: The investigator asked 3 simple questions: What is your name? Where are you from? Why are you here today?
2. Ask the subject to make a natural and a forced smile.
3. Pronunciation by the investigator of 3 funny sentences, asking the subject to repeat them. These included two French tongue twisters: “Les chaussettes de l’archiduchesse sont-elles sèches ou archi-sèches?” “Tes laitues naissent-elles? Yes mes laitues naissent”. Two English tongue twisters could also be used: “She sells sea-shells on the sea-shore of Seychelles”, and “If Peter Piper picked a peck of pickled peppers, how many pickled peppers would Peter Piper pick?”

To finish, the last sentence was “Pretend to be happy to see me and have a good time” in order to get a smile or even a spontaneous laugh in order to uncover the maximum of visible gum.

This scenography started again from step 2 after each change of position (left side, ¾ left, front, ¾ right, right side positions).

### 2.4. Editing of the Videographs

The aim of the editing was to select the time periods corresponding to the analyzed criteria. Arbitrarily, a duration of 45 s was chosen for the complete video sequence (Supplementary Materials).

### 2.5. Data Collection

The videographs were anonymized and numbered chronologically from 001 to 065.

**Figure 2:**
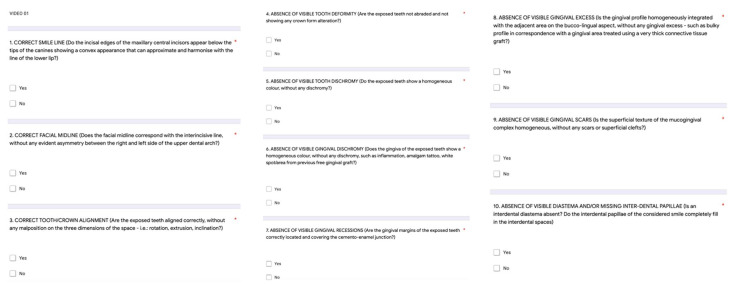
Smile Esthetic Index questionnaire (Rotundo et al., 2015) customized on Google forms for SEI data collection. The score (with a maximum of 10 points) was calculated as the sum of the marks attributed to each of the 10 answers (yes = 1 point; no = 0 point).

The questionnaires were available on an online customized Google form (Figure 2) that allowed data collection.

The score (with a maximum of 10 points) was calculated as the sum of the marks attributed to each of the 10 answers: yes = 1 point; no = 0 point.

First, a training phase consisted in watching videographs and understanding each question of the questionnaire by the 3 raters together. Then, separately, each rater watched each video as many times as they wanted with the possibility to stop and go back, and filled in the online Google form.

The calculation of the intra-rater agreement consisted of each rater filling in the online questionnaire on Google forms again, one week after the first evaluation. In order to avoid bias, the order of viewing (and their numbering) of the videographs of the 2 consecutive viewings one week apart was determined using 2 online randomization tables (https://www.randomizer.org/ (accessed on 1 July 2019)) to ensure that the rater did not remember his previous answers.

### 2.6. Statistical Analysis

The statistical analysis was carried out using XLstat software, version 3.1. (Addinsoft^®^, Bordeaux, France).

Intra-rater agreements of each rater were calculated for each of the 10 questions using Cohen’s Kappa tests. In addition, a Fleiss’ Kappa test was performed to obtain the inter-rater agreement for each of the 10 questions.

Furthermore, the Cohen’s Kappa results were interpreted according to Landis and Koch’s scale [19].

Statistically significant difference was set at a *p*-value of 0.05 (*p* < 0.05).

## 3. Results

Our sample consisted of 24 men and 41 women, i.e., 36% men and 64% women. The age of the subjects ranged from 21 to 74 years (mean age 33.0 ± 11.3 years). The average scores of the three raters ranged from 6.51 to 6.72 with an overall mean of 6.64 (Table 1).

The intra-rater agreement rates of the three raters were 0.73, 0.64 and 0.67 (Table 2), which is considered to be a substantial agreement according to Landis & Koch (Table 3) [19].

For the 10 SEI questions, inter-rater variations ranged from 0.31 to 0.90 (*p* < 0.001). The lowest agreement was obtained for the question assessing the absence of visible excessive gingiva (Fleiss’ Kappa = 0.31), whereas the highest agreement (Fleiss’ Kappa = 0.90) was obtained for the one assessing the absence of diastema and/or missing inter-dental papilla (Table 4).

## 4. Discussion

The analysis of the smile by the periodontist is a key step in better understanding the patient’s expectations as well as a tool for diagnosis and establishment of therapeutic proposals. The aesthetics of the smile are based on a global harmony between the labial frame, the gingival frame and the teeth [20]. Beyond its subjective character, the beauty of a smile is evaluated by (a) facial references, (b) criteria related to the teeth and (c) the periodontium such as the smile line, the shape and the colour of the teeth, and the gingival contour [21,22,23]. Currently, the Smile Esthetic Index, based on the evaluation of 10 variables, is the only reliable and reproducible method to objectively quantify the aesthetic value of a smile [4]. Namely, the absence or presence of each of the 10 variables are scored (0 or 1) and the sum of the 10 scores corresponds to the SEI of the subject (from 0, very bad to 10, very good). To date, the SEI has only been validated on photographs, whereas the recording of short videographs seems to allow a more accurate appreciation of the visibility of the periodontium during the smile and thus a better aesthetic analysis [18].

Our sample consisted of 24 men and 41 women, i.e., 36% men and 64% women. In our study there were more women than men; in fact, our method of recruitment over time reflects the higher proportion of women coming to our periodontal department as shown in a recent study [24].

Our results showed a mean inter-rater agreement of 0.59 (0.31–0.90) and a mean intra-rater agreement of 0.68 (0.64–0.73), demonstrating the feasibility and reproducibility of quantifying the aesthetic value of a smile from videographs. In the original photographic study, Rotundo et al. achieved a mean inter-rater reproducibility of 0.45 (0.17–0.75) based on Fleiss’ Kappa for SEI assessment [4]. Our higher agreement results may be partly related to the fact that our study had only three raters whereas Rotundo et al. had ten. In addition, our three raters were exclusively periodontists, whereas the raters in the Rotundo et al. study were more heterogeneous, including periodontists, general dentists, orthodontists and restorative dentists.

In our study, the lowest inter-rater agreement was obtained for the question “absence of visible excessive gingiva” (Fleiss’ Kappa = 0.31). In the Rotundo et al. study, the lowest inter-rater agreement was obtained for the question “absence of visible scar” (Fleiss’ Kappa = 0.17) [4]. This difference can be explained by the subjectivity of the notion of “excessive” gingiva in our study. On the other hand, Rotundo et al. pointed out the quality of the images used and the fact that only 5% of these images showed the presence of a scar as possible explanations for the low inter-rater agreement. The highest inter-rater agreement was obtained for the question “absence of diastema and/or missing inter-dental papilla” in our study as in the Rotundo et al. study (Fleiss’ Kappa = 0.90 and 0.75, respectively). According to Rotundo et al. and our results, we can speculate that videographs can be used for evaluating the SEI as photographs are [4].

Many studies have been conducted in order to determine the parameters of an ideal smile [4,25]. Different aesthetic scores have been proposed and validated using photographs but none, to our knowledge, has been validated using videographs. However, videography is the only means of capturing the amplitudes of movement of the lips as well as the different angles of view of the gingiva, which are very difficult to obtain on a single photograph [26]. Thus, when comparing the diagnostic value of photography and videography in the evaluation of a smile, Walder et al. observed a clear preference of the raters for videography, emphasizing the fact that it gives more information than still images [13]. In addition, photographs of the smile taken on different days exhibited differences in 80% of cases. Videography thus seems to allow a more faithful and reproducible evaluation of the aesthetic criteria of a smile. Chaves et al. evaluated the influence of a maxillary midline diastema on the aesthetic perception of a panel including orthodontists, restorative and prosthetic dentists, and laypersons, using videography [17]. The reliability of their method was confirmed by an intra-class correlation value of 0.81 for orthodontists, 0.73 for restorative and prosthetic specialists, and 0.71 for laypersons.

Videography seems to be a particularly relevant tool in the evaluation of smile aesthetics because it captures a set of mimics rarely visible on simple photographs taken in front of a practitioner [27]. Furthermore, the patient is accustomed to shooting with a smartphone in his everyday life. Thus, it is easier to relax the patient in front of a smartphone than in front of the lens of a more impressive professional camera which creates more “medical” distance and tenses the mimics. The strength of our study lies in the simplicity of the setting for recording videos of patients’ smiles. Furthermore, the use of videographs should be one of the pedagogical tools for the training of future dentists [28] in order to teach them how to preserve or restore the smile of their patient.

The main limit of this study is the limited number of only three raters, which includes the investigator who recorded and edited the videographs. This caveat will be corrected in a future study.

We did not correlate our objective evaluation of the SEI with the patient’s subjective evaluation and with the oral health criterion [29,30]. This could also be addressed in a future study. We also observed that the maximum smile in these videographs revealed a larger amount of gingiva than the forced smile picture.

It would be interesting to quantify the amount of gingival visibility on the videographs to increase the accuracy of diagnosis and evaluation of aesthetic results. Following this, the gingival aesthetics during the smile before and after periodontal plastic surgery could be compared.

## 5. Conclusions

Within the limits of this study, we have demonstrated that it is possible to reproducibly evaluate an aesthetic score (SEI) on videographs which was previously validated on photographs.

It would be important to correlate the oral health criterion to the assessment of smile aesthetics for future research. We can also conclude that it is possible to simply make short videographs of the smile and laughter of our patients with a smartphone associated with an affordable investment in materials: a Smile Lite and an adjustable tripod. The advantage of the videograph over the photograph is that it gives us more precise information on the visibility of the teeth and periodontium during a natural mimicry. Moreover, if necessary, the pause/capture of some sequences allow us to choose the most relevant snapshot(s) to evaluate the different aesthetic criteria.

## Figures and Tables

**Table 1:** Overall mean scores of each rater for the 10 questions.

Question No.	Rater 1	Rater 2	Rater 3
1 (correct smile line)	26	32	29
2 (correct face midline)	52	51	51
3 (correct tooth/crown alignment)	25	22	20
4 (absence of visible tooth deformity)	30	38	39
5 (absence of visible tooth dyschromy)	46	48	45
6 (absence of visible gingival dyschromy)	51	53	52
7 (absence of visible gingival recessions)	39	39	42
8 (absence of visible gingival excess)	55	53	55
9 (absence of gingival visible scars)	64	64	64
10 (absence of visible diastema and/or missing inter-dental papillae)	37	39	41
Mean overall scores on 10 questions	6.72 ± 0.07	6.51 ± 0.12	6.69 ± 0.08

**Table 2:** Intra-rater agreement among the three raters for the 10 questions.

Questionnaire	Rater 1	Rater 2	Rater 3
Mean	0.73	0.64	0.67
Min	0.46	0.30	0.42
Max	1.00	0.99	0.98

**Table 3:** Assessment of the level of agreement according to Landis and Koch [19].

Strength of Agreement	Kappa Values
Poor	<0.00
Slight	0.00–0.20
Fair	0.21–0.40
Moderate	0.41–0.60
Substantial	0.61–0.80
Perfect	0.81–1.00

**Table 4:** Inter-rater agreement by Fleiss’ Kappa test for each of the 10 SEI questions among the three raters.

Questions	Fleiss’ Kappa	*p*-Value
1 (correct smile line)	0.72	<0.0001
2 (correct face midline)	0.51	<0.0001
3 (correct tooth/crown alignment)	0.55	<0.0001
4 (absence of visible tooth deformity)	0.47	<0.0001
5 (absence of visible tooth dyschromy)	0.73	<0.0001
6 (absence of visible gingival dyschromy)	0.65	<0.0001
7 (absence of visible gingival recessions)	0.69	<0.0001
8 (absence of visible gingival excess)	0.31	<0.0001
9 (absence of gingival visible scars)	0.39	<0.0001
10 (absence of visible diastema and/or missing inter-dental papillae)	0.90	<0.0001

## Data Availability

The data presented in this study are available in Supplementary Materials.

## Supplementary Materials

The following supporting information can be downloaded at: https://www.mdpi.com/article/10.3390/dj10050087/s1; Video S1: Videography 1; Video S2: Videography 2.
